# Gender Differences in Pain: Examining Explanations for the Association

**DOI:** 10.1093/geroni/igab046.3228

**Published:** 2021-12-17

**Authors:** Cherish Michael, Anne Barrett

**Affiliations:** Florida State University, Tallahassee, Florida, United States

## Abstract

Physical pain is a gendered experience: Women report higher levels of it than do men. This pattern may stem from differences in experiences of the body. Women are socialized to be attentive to its functioning, appearance, and sensations, while men are discouraged from paying much attention to their bodies. Little is known, however, about the precise social and economic pathways leading to gender differences in pain, especially in middle and later life when pain is most prevalent. We examine this issue using data from Wave 3 of Midlife in the United States (2013-2014). We consider four possible explanations for women’s more frequent reports of pain: economic security, physical and mental health, social relationships, and discrimination. Results indicate that women are more likely than men to report experiencing chronic pain, as well as greater effects of it on their everyday lives. However, only two of the explanations contributed to explaining this association. Economic security and physical and mental health accounted for substantial portions of the association between gender and pain – 57 and 73 percent, respectively. In contrast, no mediating role was observed for either women’s social relationships, in particular the greater strain they experience in them, or their more frequent reports of everyday and lifetime discrimination. The final model including all the possible explanations revealed that gender was no longer significant, suggesting that middle-aged and older women’s greater pain is explained by their worse health and economic circumstances.

